# Development and Characterization of Pseudotype-based Microneutralization Assay to Assess Functionality of Anti-rabies Protein G Antibodies

**DOI:** 10.12688/f1000research.178284.2

**Published:** 2026-10-06

**Authors:** Antonelli R#, Vezzani G#, Guerrini N, Giovagnoli V, Dapporto F, Iturriza M, Loomis R, Montomoli E, Rossi O, Molesti E, Manenti A

**Affiliations:** 1 Vismederi, siena, 53100, Italy; 2 GVGH, GSK Vaccines Institute for Global Health, Siena, Italy; 3 Department of Public Health, Experimental and Forensic Medicine, University of Pavia, Pavia, 27100, Italy; 4 Department of Molecular and Developmental Medicine, University of Siena, Siena, 53100, Italy

**Keywords:** neutralization, rabies, vaccines, protein G, functional

## Abstract

**Background:**

Rabies virus (RABV) is a neurotropic zoonotic pathogen responsible for an estimated 60,000 human deaths annually. Despite widespread vaccination programs, rabies remains a substantial public health burden, particularly in regions with limited access to post-exposure prophylaxis (PEP). Standard serological assays for evaluating rabies vaccine immunogenicity, including the Rapid Fluorescent Focus Inhibition Test (RFFIT) and the Fluorescent Antibody Virus Neutralization (FAVN) test, require live virus handling under biosafety level 3 (BSL-3) conditions, limiting their scalability and broader implementation.

**Methods:**

We developed and characterized a pseudotype-based microneutralization assay using a lentiviral HIV-1 backbone to generate pseudotyped viral particles expressing glycoproteins from three antigenically distinct rabies virus strains: CTN-1 V5, CVS-11, and Pasteur. Neutralization assays were performed under BSL-2 conditions, and assay performance was evaluated in terms of sensitivity, specificity, and suitability for high-throughput applications.

**Results:**

The pseudotype-based assay enabled sensitive and reproducible detection of rabies virus–neutralizing antibodies across the three glycoprotein variants. The platform demonstrated robust performance under BSL-2 containment, supporting efficient and scalable testing. Inclusion of antigenically distinct strains broadened detection capacity and improved the assessment of strain-specific neutralizing responses.

**Conclusions:**

This pseudotype-based microneutralization assay represents a safe, flexible, and scalable alternative to conventional live-virus neutralization tests. By enabling high-throughput rabies serology under BSL-2 conditions, this platform supports vaccine evaluation and seroepidemiological surveillance, particularly in settings where BSL-3 infrastructure is limited.

## Introduction

Rabies virus (RABV) is a neurotropic, zoonotic pathogen that causes an almost in-variably fatal encephalitic disease if left untreated. Responsible for approximately 60,000 human deaths annually, rabies remains a significant global health burden, particularly in regions with limited access to post-exposure prophylaxis (PEP). ^1, 2^ The virus is primarily transmitted to humans through the bite of infected dogs, which account for 99% of human cases worldwide. ^3^ Following exposure, RABV spreads from the peripheral to the central nervous system, resulting in acute encephalomyelitis and, in nearly all cases, death. ^4^

RABV is a non-segmented, negative-sense RNA virus of the Rhabdoviridae family. Its genome encodes five proteins: nucleoprotein (N), phosphoprotein (P), matrix protein (M), glycoprotein (G), and the large RNA-dependent RNA polymerase (L). Among these, the surface-exposed G protein is the sole target of virus-neutralizing antibodies and is thus central to vaccine and therapeutic development. ^5^ Com-prising 524 amino acids, the G protein includes an ectodomain that is highly im-munogenic. However, over 10% sequence divergence among natural strains poses challenges for monoclonal antibody development, as certain variants can escape neutralization by antibodies currently in use or under clinical investigation. ^6^

Vaccination remains the most effective strategy for rabies prevention. Although nerve tissue-derived vaccines are still in use in some countries, the World Health Organization (WHO) recommends their replacement with safer and more immu-nogenic cell culture-based vaccines. Intradermal administration of these modern vaccines has been shown to be both cost-effective and immunologically equivalent to intramuscular injection, particularly in resource-limited settings. ^7^ To further strengthen the effective use of rabies vaccines, the WHO launched in 2022 an in-ternational collaborative study (WHO/BS/2022.2435) involving laboratories worldwide to evaluate the candidate 3rd International Standard (IS) for Rabies Immunoglobulin, designed to support the standardization of potency assays measuring neutralizing antibody levels in therapeutic human rabies immuno-globulin (HRIG) and serology assays assessing antibody levels in human and animal serum. ^8^

However, despite global efforts to improve vaccine coverage, there remains a lack of objective and effective methods to rigorously evaluate differences in protective efficacy among rabies vaccines. ^9^ In this context, accurate and scalable quantification of virus-neutralizing antibodies is essential for assessing vaccine-induced immunity, informing immunization strategies, and monitoring seroconversion in both clinical and preclinical studies. ^10^

Traditional methods such as the Rapid Fluorescent Focus Inhibition Test (RFFIT) and the Fluorescent Antibody Virus Neutralization (FAVN) test are considered the gold standards in rabies serology. However, their reliance on live, pathogenic virus requires biosafety level 3 (BSL-3) containment, limiting their scalability and acces-sibility, especially in low-resource settings. ^11^

To address these limitations, pseudotyped virus-based microneutralization assays have emerged as a promising alternative. These assays utilize replication-incompetent viruses engineered to express the main antigenic protein of interest on their surface, allowing for sensitive, high-throughput virus neutralization assay detection under BSL-2 conditions still preserving antigenic relevance to circulating strains, thus being fit-for-purpose to asses neutralizing ability of antibod-ies induced in vaccine studies. ^12^

In this study, using as starting point the study of Wright and colleagues, ^13^ we setup a pseudotypes microneutralization assay using an HIV-based platform to produce three pseudotyped particles (PV), each of them bearing glycoproteins from three antigenically distinct rabies virus strains: CTN-1 V5 (a widely used Chinese vaccine strain which was isolated and stored by The National Institute of Food and Drug Control of China) ^14^; CVS-11, a standard challenge strain for potency testing ^15^; and the Pasteur strain, commonly used in both human and veterinary vaccines. ^16^

The characterization of the microneutralization was performed with both the WHO 3rd International Standard 19/244, and a panel of four different monoclonal antibodies: RVA122, ^16^ 17C7, ^17^ 523–11, ^18^ and 1112–1. ^19^ The use of this panel can allow the evaluation of neutralizing antibody responses across a relatively broad range of rabies lineages. The data presented demonstrate the utility of this assay as alternative and robust platform for rabies serology, supporting its use for both vaccine evaluation and seroepidemiologic surveillanc.

## Materials and methods

### Cell culture

Human embryonic kidney 293 T/17 cells (HEK293T/17; ATCC, CRL-11268) were utilized for the production and titration of rabies virus glycoprotein (G) variants in a pseudotype-based microneutralization (MN) assay. Cells were maintained in Dulbecco’s Modified Eagle Medium (DMEM) containing high glucose and sodium pyruvate (Gibco, Cat. No. 11995073), supplemented with 10% heat-inactivated fetal bovine serum (FBS; South American origin, EU-certified; Euroclone, Cat. No. ECS5000LH) and 1% penicillin-streptomycin (PenStrep; Merck, Cat. No. P4333). Cultures were incubated at 37°C in a humidified 5% CO _2_ atmosphere.

### Plasmid constructs and transduction efficiency

Codon-optimized sequences encoding the G proteins of rabies virus strains CVS-11 (GenBank: EU352767.1), ^13^ CTN1V5 (GenBank: JN234418.1), and Pasteur (GenBank: NP_056796.1) were synthesized by GenScript. These sequences were inserted into the pI.18 vector, a high-copy plasmid featuring an ampicillin resistance gene and driven by the human cytomegalovirus (hCMV) promoter, enabling robust expression in mammalian cells. ^21^ For pseudoviruses packaging, the envelope-deficient HIV-1 backbone plasmid pNL4–3.Luc.R-E (repository reference ARP2128 obtained from the NIH AIDS Reagent Program) was employed. This construct expresses luciferase and lacks a functional envelope gene. Each G construct was co-transfected into HEK293/17 cells together with the pNL4–3.Luc.R ^−^E ^−^ plasmid, which contains a firefly luciferase reporter gene. To identify the optimal conditions for high-titre pseudoviruses (PV) production, different plasmid ratios were tested (4.5 μg pNL4–3.Luc.R ^−^E ^−^ with 4.5 μg G plasmid; 6 μg pNL4–3.Luc.R ^−^E ^−^ with 4.5 μg G plasmid) along with varying transfection incubation times (24, 48, and 72 h); at those time point, supernatants were collected and titrated. Transduction efficiency was quantified by measuring luciferase activity, expressed as relative light units per millilitre (RLU/mL).

### Pseudotyped particles generation

To produce pseudoviruses, HEK293T/17 cells (4.5 × 10 ^6^) were plated in 10 cm dishes. After 24 hours, cells were transfected with a mixture containing 9 μg of pNL4–3.Luc.R-E and 9 μg of the G protein-encoding plasmid, using 34 μL of EndoFectin™-Lenti transfection reagent (GeneCopoeia, Cat. No. EF001) diluted in 500 μL of Opti-MEM™ Reduced Serum Medium (Gibco, Cat. No. 31985–070). Six hours post-transfection, the culture medium was replaced with 8 mL of fresh DMEM. After 72 hours, the supernatant containing the pseudotyped viral particles was collected, clarified by low-speed centrifugation, passed through 0.45 μm syringe filters, and stored at −80°C until further use.

### Monoclonal antibodies and serum samples

Four human monoclonal antibodies (mAbs), previously described to neutralize Rabies virus, were selected for this work: RVA122, ^17^ 17C7, ^18^ 523–11, ^19^ and 1112–1. ^20^ Starting from variable and light chain sequences, all mAbs were cloned in pcDNA3.4 plasmids bearing in frame the constant chain of interest (CH1 for variable heavy sequence, and CK for variable light sequence). The four mAbs in IgG1 scaffold were produced by GenScript by co-transfecting the generated plasmids in CHO cells. All mAbs were checked for purity and integrity by size exclusion chromatography, and resuspended in PBS at the final concentration of 0.5 mg/ml.

WHO 3 ^rd^ international standard (NIBSC 19/244) was purchased from MHRA (formerly NIBSC). Immunoglobulin-depleted serum (BBI Solutions, Catalog No. SF505–2) was used as negative matrix. To assess specificity, the Golden West serum (W425623105626) and the anti-Chikungunya virus E2 antibody (CHK-265; Cat. No. HY-P990825, MedChemExpress) at 0.25 μg/starting concentration were evaluated (Extended Data). All mAbs and international standard were stored at −80°C upon use.

### Pseudoviruses titration

Titration of pseudotyped viruses was carried out in 96-well plates. At first, 50 μL of DMEM was added to each well, except those in row A, which received 100 μL of the pseudoviruses preparation. Serial two-fold dilutions were performed from row A to row G. Subsequently, 1 × 10 ^5^ HEK293T/17 cells were added to each well. Following a 72-hour incubation at 37°C with 5% CO _2_, 50 μL of Bright-Glo™ Luciferase Assay Reagent (Promega, Cat. No. E2620) was added per well. The plates were shaken for 5 minutes at 400 RPM using a microplate shaker (Fisherbrand, Cat. No. 88861024). Luminescence was then measured using the GloMax ^®^ Plate Reader (Promega) under the following settings: endpoint luminescence mode, single label detection, 700 nm, with a 1000 ms integration time.

### Pseudotype based microneutralization assay

The pseudotype-based microneutralization assay (PBNA) was performed in a 96-well plate format. The working solution of RABV pseudotypes was adjusted to yield 5 × 10 ^7^ relative luminescence units (RLU), corresponding to an input of 5 × 10 ^5^ RLU per well. 90 μL of DMEM were added to column one and 50 μL were added to the other wells. To column 1 were added 10 μL of serum samples (WHO 3rd International Standard 19/244 or monoclonal RABV antibodies at 0.25 μg/starting concentration) or immunoglobulins depleted serum (BBI Solutions, Catalog number: SF505–2), used as negative control. Serial two-fold dilutions were performed from column 1 to 10. Subsequently, 50 μL of pseudotype working solution were added to each well up to column 10, and to column 12 from raw A to H (pseudotype control wells). After incubating the plate containing the mix pseudotypes-serum for 1 hour at 37°C with 5% CO _2_, 1 × 10 ^5^ HEK293T/17 cells were added to each well. After 72 hours, 50 μL of Bright-Glo Luciferase Assay System were dispensed in each well and the plate was shaken for 5 minutes at 400 rpm in a dark environment (Fisherbrand, Cat. No. 88861024). Plate readout was performed with a luminometer (Perkin Elmer; Victor Nivo).

### Data analysis

Pseudoviruses titres were calculated in Microsoft Excel™ and expressed as relative luminescence units per millilitre (RLU/mL) using the formula:

Titre=((RLU−CCcontrol signal)×dilution factor))/volume of pseudotypesperwell(ml))

PBNA results were analysed using Microsoft Excel (Microsoft Corporation, Redmond, WA, USA) and GraphPad Prism version 8.4 (GraphPad Software, San Diego, CA, USA). Microsoft Excel was used to visualize the raw data, as the software automatically opened the comma-separated values (CSV) files generated by the GloMax ^®^ Multi Detection System luminometer. Excel was also used to calculate the average values of the pseudotype-only control wells (PP controls) and the cell-only wells (CC controls), as well as the logarithmic dilution factors using the LOG10 function.

Subsequent analysis was performed in GraphPad Prism. An XY table was created using the plotting a single Y value for each point. Raw values were normalized to the mean of the PP (pseudotype-only) and CC (cell-only) controls using the Analyze and then Normalize function. Neutralization curves were fitted using nonlinear regression analysis (log [inhibitor] vs. normalized response, variable slope), and half-maximal inhibitory concentrations (IC _50_) were calculated. The detailed step-by-step protocol for pseudotype-based neutralization assays is described by Ferrara and Temperton 2018. Final titres were expressed as the dilution interval corresponding to the IC _50_, in accordance with the method described by Hyseni et al. ^22, 23^

### Precision

Precision is used to define the amount of scatter between several measurements obtained from multiple testing of the same sample under the standardized conditions. Precision was evaluated in terms of repeatability (intra-assay variation) and intermediate precision (inter-assay variation). The sample dilutions were tested in duplicate in a two-fold dilution scheme, from a starting dilution of 1:2000 for international standard, 1:100 for negative matrix, and from 0.25 μg/mL for mAbs by two different operators, on three different days. Log-transformed IC _50_ results from the three runs were analysed with Minitab 18 (Minitab, LLC, State College, PA, USA) applying a Mixed Effects Model considering the day as random factor to determine the repeatability (CV% R) and intermediate precision (CV% IP) of the assay. ^24^

## Results

To develop robust pseudotype virus neutralization assay for rabies that could be handled in biosafety level 2 containment, a pseudotype based microneutralization assay has been developed using the WHO 3rd International Standard and immunoglobulin-depleted serum included as test or control samples, respectively ( Figure 1).

**Figure 1.**
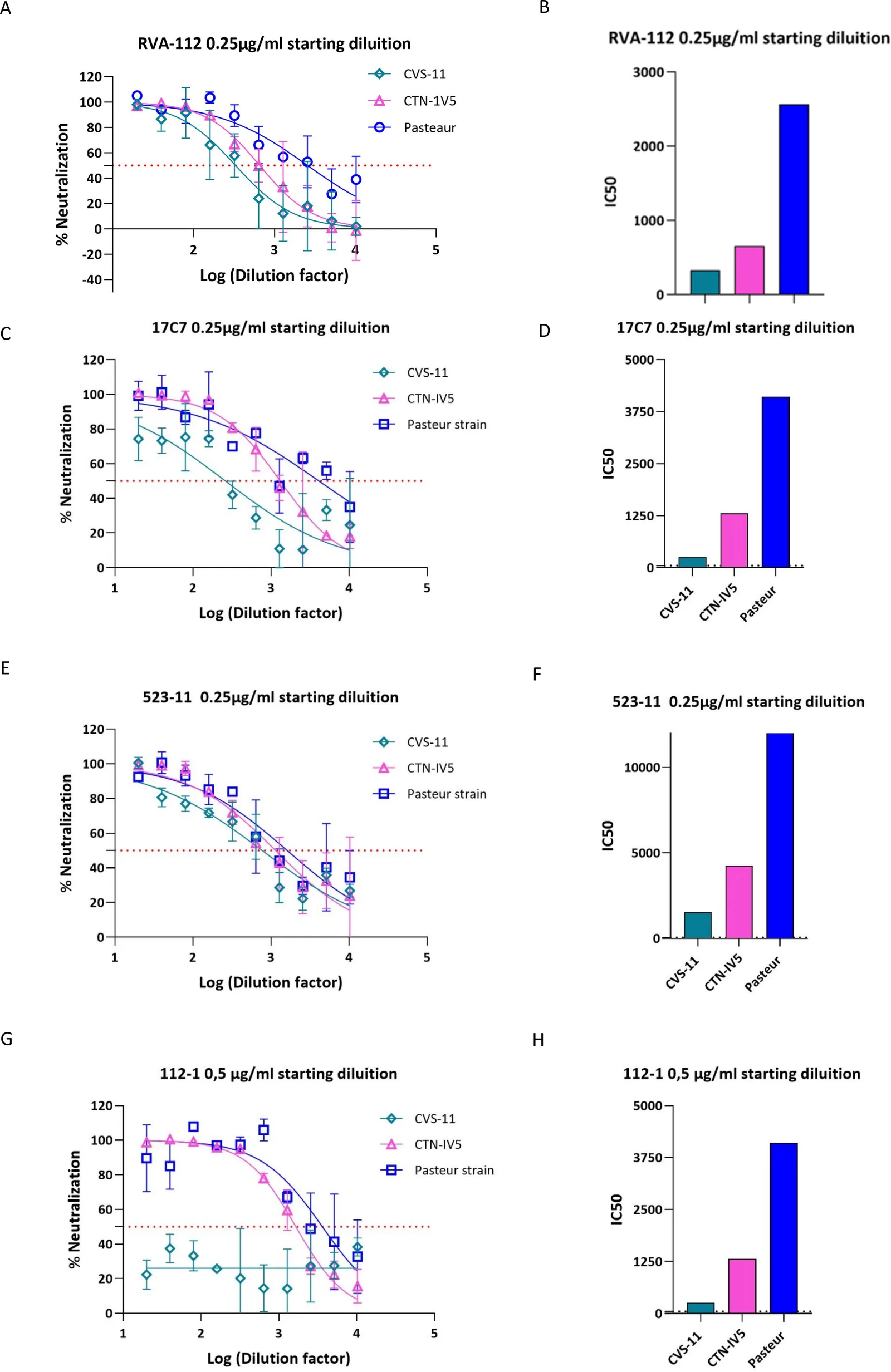
Assay workflow. From top left corner of the image, following clockwise order, the packaging vector pNL4–3.Luc.R ^−^E ^−^, encoding the firefly luciferase reporter gene is co-transfected with p.i18G RABV strain plasmids into HEK293T/17 cells. Pseudotype particles are assembled in the cells and released at the plasma membrane, yielding pseudoviruses bearing the desired glycoproteins and the luciferase reporter. Culture supernatants are collected 72 h post-transfection and filtered through a 0.45 μm filter. The resulting pseudoviruses are titrated to determine particle concentration. For the pseudotype microneutralization assay, 5 × 10 ^5^ PV per well are incubated for 1 h with serial dilutions of serum or monoclonal antibodies (mAbs) according to the plate scheme, followed by the addition of 1 × 10 ^5^ HEK293T/17 cells per well. After 72 h of incubation, cells are lysed with Bright-Glo reagent and luminescence was measured using a Glomax luminometer. Titres are expressed as relative luminescence units per millilitre (RLU/mL) after normalization to the means of pseudotype-only (PP) and cell-only (CC) controls. Neutralization curves are generated by nonlinear regression (log [inhibitor] vs. normalized response, variable slope), and half-maximal inhibitory concentrations (IC _50_) are calculated.

The coding sequences of the glycoprotein (G) from three rabies virus strains CVS-11, CTN1V5, and Pasteur were synthesized and cloned into the pI.18 expression vector and co-transfected into HEK293/17 cells together with the pNL4–3.Luc.R ^−^E ^−^ plasmid, which contains a firefly luciferase reporter gene. Optimization experiments identified that high titre pseudoviruses production was achieved using 9 μg of each plasmid per transfection and an incubation period of 72 h. In titration experiments, an input of 5 × 10 ^5^ RLU per well was determined to be optimal, providing a high dynamic range that allows clear discrimination between neutralizing and non-neutralizing samples, thus ensuring high assay sensitivity and resolution.

In the microneutralization assay, all samples were tested in duplicate and serially diluted across columns 1–10. Column 11 was left empty, while column 12 was used for controls: the first four wells (12A–12D) contained cell-only controls, and the last four wells (12E–12H) contained pseudotype-only controls. 5 × 10 ^5^ RLU pseudoviruses particles were added per well and incubated with the serum dilutions for 1 hour at 37°C. Following this incubation, HEK293T target cells were added at a density of 1 × 10 ^5^ cells per well, and plates were incubated for 72 hours. Afterwards luciferase activity was measured, obtaining RLU/mL for each point of the dilution series; the RLU/mL versus Log transformed dilutions were used to fit a 4PL Logistic curve and calculate the IC _50_.

### Qualification of pseudoneutralization assays targeting rabies protein G

Assay performance was evaluated using pseudotype microneutralization assays with four monoclonal neutralizing antibodies targeting distinct epitopes on the rabies virus glycoprotein (RABV-G), the international standard serum (IS), and an IgG-depleted matrix. The panel included 17C7, which recognizes a conformational epitope encompassing antigenic site III ^18^ on RABV-G; RVA122, which interacts with residues in domain I and domain III; 1112–1, ^19^ which targets antigenic site II, a conformational epitope located in the central domain of the prefusion glycoprotein trimer; and 523–11, which also recognizes antigenic site II on RABV-G. ^17^ To evaluate the repeatability and intermediate precision of the assay, the IS and all mAbs were tested in 3 different analytical sessions, performed by two operators with two replicates per session ( Figure 2). ^25^

**Figure 2.**
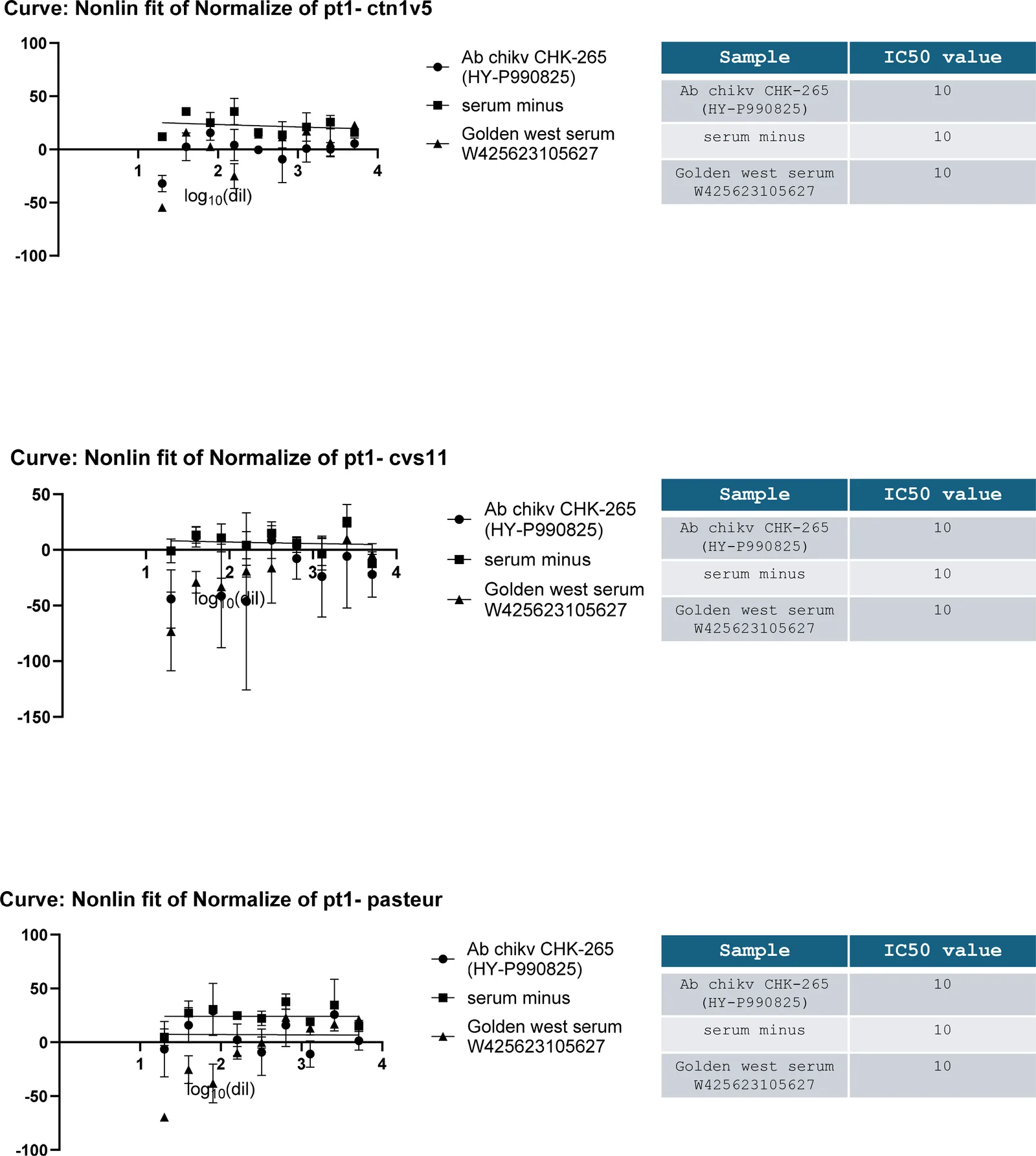
Assay precision: IC50 obtained for the mAbs (left panels, or sera (right panels) are reported against the three different protein G pseudotypes strains (from top to bottom, CVS-11, Pasteur, and CTN1). Values reported in the same colour are obtained in the same day, and full-empty symbols correspond to two different operators.

Across all rabies protein G pseudotype strains tested, the logarithm of coefficient of variance (Log CV%) values remained within predefined acceptance criteria, with ≤20% for repeatability and ≤ 30% for intermediate precision, for both the IS and all monoclonal antibodies ( Table 1). Apart for 1112–1 mAb on the strain CVS-11, CV% of all the other mAbs on all the tested strains resulted to be inferior to 10%.

**Table 1.** Assay precision.

	CVS11 log CV%		Pasteur log CV%		CTN1v5 log CV%	
	Repeatability	Intermediate Precision	Repeatability	Intermediate Precision	Repeatability	Intermediate Precision
RVA-122	6,95	6,95	4,13	5,92	3,36	6,38
17C7	8,02	9,91	4,50	7,77	3,41	5,53
523–11	4,61	6,44	5,09	6,70	1,43	6.91
1112–1	10,12	23,88	3,17	5,10	2,79	4,34
19/244	4,41	4,51	1,62	3,76	1,85	2,84

In the table are reported the CV% values for the four mAbs tested and the international standard 19/244 on the three different strains (from left to right: CVS11, Pasteur, and CTN1v5 Rabies strains). For each strain, repeatability is reported on the left side of the column, while intermediate precision on the right one.

## Discussion

According to the WHO, rabies remains one of the 21 neglected diseases in developing countries. ^26^ Despite over 29 million rabies vaccinations being administered annually, globally one person dies from rabies every nine minutes, with almost half of these deaths occurring in children. ^27^ The current reliance on only three WHO-prequalified human rabies vaccines, RABIVAX-S, ^25^ VaxiRab N, ^28^ VERORAB, ^29^ raises concerns about the sufficiency of vaccine supply and equitable access worldwide. The recent rabies outbreak in Timor-Leste, which resulted in four confirmed human deaths, and over 1,400 dog bite incidents between March 2024 and June 2025, starkly illustrates the persistent risk of rabies transmission, particularly in regions with fragile health systems. ^30^ This outbreak exposes critical gaps in rabies surveillance, post-exposure prophylaxis (PEP) availability, and community awareness, especially as the virus spreads into previously unaffected areas. Recent advances in vaccine technology including modified live, recombinant, and nucleic acid-based vaccines offer promising improvements in immunogenicity, safety, and accessibility. ^31^

In parallel, the development of international biological standards and appropriately validated assays is critical to accurately assess vaccine-induced immune responses and to promote standardization, transparency, and comparability across rabies vaccine candidates. A standardized neutralization assay facilitates the selection and down-selection of vaccine candidates for clinical development. Being the only protein exposed on the viral surface, the G protein is central to vaccine design, eliciting both humoral and cell-mediated immune responses. Indeed, the rabies virus G protein (RABV-G) is the primary target for neutralizing antibodies and serves as a key correlate of protection, with vaccine-elicited antibodies predominantly targeting immunodominant epitopes such as site III, which benefits from favourable physicochemical properties for antibody binding. ^32^ Importantly, the World Health Organization (WHO) has established that a neutralizing antibody titre of ≥0.5 IU/mL against the G protein represents the minimum protective threshold, serving as a reference for vaccine efficacy and standardization of immunological assays. ^33^

Wright and colleagues were the first to propose pseudotype-based neutralisation assay (PBNAs) as an alternative assay for detecting rabies virus (RABV) antibodies, demonstrating their utility for sensitive and specific measurement of neutralizing antibody responses. ^13^ Building upon this foundation, Nie and colleagues developed a novel platform capable of generating high-titer rabies pseudoviruses for both in vitro and in vivo analyses of rabies-specific immunoglobulins and vaccine-induced immunogenicity. ^34^ Therefore, PBNA represents a reliable instrument that can serve as a surrogate for RABV live viruses, as those viruses necessitate high-containment facilities and skilled operators. To further corroborated this, we pseudotyped G proteins of three different strains (CVS-11, CTN1V5, and Pasteur), and optimized their production in HEK293T/17 cells before assessing performance of the assay. The higher pseudoviruses titre observed for the CTN1V5 strain, compared to CVS-11 and Pasteur, highlights how strain-specific differences in the G protein aminoacidic sequence could provide insights into receptor-virus binding affinity, consistent with previous reports emphasizing the importance of glycoprotein conformational integrity on viral entry efficiency. ^35, 36^ Indeed, using conformation-dependent mAbs targeting different epitopes (either in one specific domain or, in one case, covering two distinct ones) we confirmed the correct assembly of Rabies glycoprotein G on the surface of the generated paseudoparticles harbouring RABVG from three different strains.

In the current work, we used the MHRA 3rd International Standard (IS) for rabies immunoglobulin, combined with four neutralizing monoclonal antibodies, to characterize the assay. The international standard provides a universal reference that enhances assay precision, reproducibility, and comparability across different laboratories and studies worldwide, which is essential for global surveillance and vaccine evaluation. Incorporating monoclonal antibodies targeting distinct viral epitopes allowed us to assess the ability of the assay to detect neutralization mediated by antibodies recognizing different regions of RABV-G and provided evidence of the functional responsiveness of the pseudotype system. This multi-epitope approach also supports the assessment of cross-protective immunity, a key factor for developing broadly effective vaccines.

Although the use of the international standard and neutralizing monoclonal antibodies provided an appropriate reference framework for assay characterization, the assay demonstrated excellent repeatability and intermediate precision, with coefficients of variation consistently within the predefined acceptance criteria, confirming its analytical reproducibility. However, due to the limited availability of a sufficiently large and representative sample panel, a formal determination of diagnostic sensitivity and specificity was beyond the scope of this study. As the assay is intended for the measurement of functional antibody responses rather than for diagnostic purposes, the validation focused on assessing its analytical performance characteristics.

Future studies including appropriate panels of well-characterized positive and negative sera, reference materials, and suitable non-neutralizing controls will be required to formally establish diagnostic and analytical performance characteristics, including sensitivity, specificity, assay cut-off values, and agreement with established reference methods such as RFFIT and FAVN. Our findings highlight several critical advantages of using pseudotype-based neutralization assays (PBNA) over conventional live virus assays such as RFFIT and FAVN. ^10^ The use of replication-incompetent pseudoviruses significantly reduces biosafety risks, enabling broader implementation in laboratories lacking high-containment facilities. Moreover, the assay’s flexibility to incorporate glycoproteins from multiple strains supports the evaluation of cross-neutralizing antibody responses, an essential feature for quantifying the breadth of vaccine-induced immunity given the antigenic diversity among circulating rabies viruses. Nevertheless, before the assay can be considered a validated alternative or complementary method for routine serological applications, further studies are needed to establish its performance against established reference assays and across a broader range of biological samples.

In this study, by employing the WHO 3rd International Standard, we standardized the pseudoviruses input to ensure optimal assay sensitivity and reproducibility. Our selected input of 5 × 10 ^5^ RLU per well for all three G protein pseudotypes (CVS-11, CTN1V5, and Pasteur) aligns closely with the range proposed by Wright et al. (2008), who first demonstrated that lentiviral pseudotypes expressing the CVS-11 glycoprotein could reliably detect neutralizing antibodies across lyssavirus species using inputs between 1 × 10 ^5^ and 1 × 10 ^6^ RLU. More recently, Swedberg et al. 2024 highlighted the potential of standardized pseudotype-based systems to harmonize neutralization measurements across laboratories and to evaluate novel vaccine platforms under lower biosafety constraints. Building on these advances, our optimized pseudoviruses input and assay configuration reinforce the precision and standardization potential of PBNA for assessing rabies virus neutralizing antibodies across diverse vaccine candidates. Future work should focus on further expanding the panel of pseudotyped strains, including emerging rabies-related lyssaviruses, to assess cross-reactive neutralization and inform universal vaccine design. Additionally, adaptation of the assay to high-throughput formats could enable large-scale serological surveillance and vaccine efficacy studies, particularly in resource-limited settings where Rabies is endemic.

In conclusion, the optimized pseudotype-based microneutralization assay described herein provides a precise and reproducible platform for the measurement of rabies virus-neutralizing antibodies. By combining enhanced safety, flexibility, and standardization, the assay represents a promising complementary approach to conventional live-virus neutralization tests, such as RFFIT and FAVN assay, which require higher biosafety containment. The pseudotype system aligns with international recommendations and reference standards, offering a practical tool for the evaluation of vaccine-induced immune responses. Furthermore, the demonstrated analytical precision and reproducibility of the assay, together with its capacity to detect neutralization mediated by monoclonal antibodies targeting distinct epitopes on the rabies virus glycoprotein (RABV-G), support its potential application in research and vaccine development. An additional advantage of the pseudotype platform is its versatility for serosurveillance studies and for investigating the impact of naturally occurring or experimentally introduced glycoprotein mutations on antibody-mediated neutralization. This capability may facilitate the monitoring of antigenic variation and the identification of mutations associated with altered neutralization sensitivity or immune selection. However, additional studies incorporating appropriately characterized panels of positive and negative sera, reference materials, and non-neutralizing controls, as well as direct comparison with established methods such as RFFIT and FAVN, will be necessary to fully characterize assay performance and assess its broader applicability. Such investigations will be important to determine whether the PBNA may ultimately serve as an alternative or complementary method for the standardized assessment of rabies virus-neutralizing antibodies in both research and public health settings.

## Ethics and consent

Ethical approval and consent were not required.

## Data Availability

The data that supports the findings of this study “Development and characterization of pseudotype-based microneutralization assay to assess functionality of anti-rabies protein G antibodies.” are available in biostudies portal Accession S-BSST2763 DOI https://doi.org/10.6019/S-BSST2763. ^25^
