# Supplementary material for: Development and Characterization of Pseudotype-based Microneutralization Assay to Assess Functionality of Anti-rabies Protein G Antibodies
Source: F1000Res. 2026 Oct 6;15:718. Originally published 2026 May 12. [Version 2] doi: 10.12688/f1000research.178284.2 (PMC13720291; doi:10.12688/f1000research.178284.2)
Supplement: Peer review history

# Reviewer report: Reviewer response for version 2

**DOI:** 10.5256/f1000research.211466.r527764

**Published:** 2026-10-29

**Authors:** Renato Mancini Astray

**Affiliations:** 1 Butantan Institute, São Paulo, Brazil

I formally approve the updated version of this article. It is really nice work.

Is the work clearly and accurately presented and does it cite the current literature?

Yes

If applicable, is the statistical analysis and its interpretation appropriate?

Yes

Are all the source data underlying the results available to ensure full reproducibility?

Yes

Is the study design appropriate and is the work technically sound?

Partly

Are the conclusions drawn adequately supported by the results?

Partly

Are sufficient details of methods and analysis provided to allow replication by others?

Yes

Reviewer Expertise:

Viral vaccines

I confirm that I have read this submission and believe that I have an appropriate level of expertise to confirm that it is of an acceptable scientific standard.

# Reviewer report: Reviewer response for version 2

**DOI:** 10.5256/f1000research.211466.r527765

**Published:** 2026-10-14

**Authors:** Raja Paramasivam

**Affiliations:** 1 Madras Veterinary College (TANUVAS), Chennai, Georgia, India

The authors have satisfactorily addressed all the comments and incorporated the necessary corrections in the revised manuscript. Therefore, the manuscript is recommended for indexing.

Is the work clearly and accurately presented and does it cite the current literature?

Yes

If applicable, is the statistical analysis and its interpretation appropriate?

Yes

Are all the source data underlying the results available to ensure full reproducibility?

Yes

Is the study design appropriate and is the work technically sound?

Yes

Are the conclusions drawn adequately supported by the results?

Yes

Are sufficient details of methods and analysis provided to allow replication by others?

Yes

Reviewer Expertise:

Experienced in rabies research and laboratory diagnosis, with specialization in molecular virology and translational diagnostics. Research experience includes molecular characterization of field rabies virus isolates, genome-based studies, development of rapid and point-of-care diagnostic assays, evaluation of recombinant monoclonal antibodies, antiviral screening, sero-surveillance and oral vaccination strategies in community dogs.

I confirm that I have read this submission and believe that I have an appropriate level of expertise to confirm that it is of an acceptable scientific standard.

# Reviewer report: Reviewer response for version 1

**DOI:** 10.5256/f1000research.196654.r511576

**Published:** 2026-09-03

**Authors:** Raja Paramasivam

**Affiliations:** 1 Madras Veterinary College (TANUVAS), Chennai, Georgia, India

Overall, the manuscript is well presented, clear, and addresses an important topic in rabies research. The methodology is described in considerable detail, and the study design and assay development are generally appropriate and of good academic relevance. I have added a few comments in the document, mainly concerning reference accuracy, as well as minor formatting, language, grammar, and typographical issues. Addressing these points would further improve the clarity and robustness of the manuscript.

 In particular, the validation of assay specificity and sensitivity should be clarified and strengthened. Although sensitivity and specificity are mentioned in the Abstract, the Results do not appear to provide sufficient data to fully support these claims, particularly with respect to specificity. The authors should clarify how these parameters were defined and assessed and provide the corresponding validation results. The absence of well-characterized negative serum samples or a non-neutralizing monoclonal antibody should also be acknowledged as a limitation, particularly given the proposed use of the assay as an alternative or complementary method to RFFIT or FAVN. If formal sensitivity and specificity were not determined, the terminology should be revised to avoid overstatement of the findings.

 The references should also be carefully checked for accuracy and appropriate citation of primary sources. In addition, the manuscript would benefit from a thorough review of formatting, language, grammar, and typographical issues throughout.

Is the work clearly and accurately presented and does it cite the current literature?

Yes

If applicable, is the statistical analysis and its interpretation appropriate?

Yes

Are all the source data underlying the results available to ensure full reproducibility?

Yes

Is the study design appropriate and is the work technically sound?

Yes

Are the conclusions drawn adequately supported by the results?

Yes

Are sufficient details of methods and analysis provided to allow replication by others?

Yes

Reviewer Expertise:

Experienced in rabies research and laboratory diagnosis, with specialization in molecular virology and translational diagnostics. Research experience includes molecular characterization of field rabies virus isolates, genome-based studies, development of rapid and point-of-care diagnostic assays, evaluation of recombinant monoclonal antibodies, antiviral screening, sero-surveillance and oral vaccination strategies in community dogs.

I confirm that I have read this submission and believe that I have an appropriate level of expertise to confirm that it is of an acceptable scientific standard, however I have significant reservations, as outlined above.

# Response

**Published:** 2026-09-25

**Authors:** Eleonora Molesti

**Affiliations:** VisMederi, Not Applicable, Siena, Italy, Italy

We thank the Reviewer for this important comment. We agree that the assessment of assay specificity and sensitivity required further clarification.

 To further assess the analytical specificity of the assay, we tested the Golden West serum (W425623105626) and an anti-Chikungunya virus E2 antibody (CHK-265; Cat. No. HY-P990825, MedChemExpress) against all three rabies virus strains included in the study. Both samples showed no detectable neutralizing activity against any of the three rabies virus strains under the experimental conditions used. The corresponding inhibition curves and experimental data have been added to the Supplementary Materials. For comparison, representative inhibition curves obtained with positive samples have also been included to illustrate the differences in IC50 values and curve profiles between positive and non-reactive samples.

 These results support the analytical specificity of the assay and indicate the absence of detectable cross-reactivity under the experimental conditions investigated. However, we acknowledge that the available dataset does not allow the establishment of a formal diagnostic cut-off or a quantitative threshold for classifying samples as positive or negative. In particular, the study did not include a sufficiently large and well-characterized panel of negative serum samples that would allow a statistically robust determination of such a threshold.

 We also acknowledge that a formal determination of diagnostic sensitivity and specificity was not performed. Consequently, we have revised the terminology throughout the manuscript to avoid overstating the performance characteristics of the assay. Specifically, references to diagnostic sensitivity and specificity have been replaced, where appropriate, by terminology referring to analytical specificity and overall assay performance.

 This limitation has been acknowledged in the revised Discussion/Limitations section. We have also clarified that further studies including appropriate negative and reference sample panels would be required to formally establish the diagnostic performance and performance characteristics of the assay.

 Accordingly, the proposed application of the assay as an alternative or complementary method to RFFIT or FAVN is now presented with appropriate caution, and the limitations of the current dataset are clearly stated in the revised manuscript.

# Reviewer report: Reviewer response for version 1

**DOI:** 10.5256/f1000research.196654.r491456

**Published:** 2026-06-22

**Authors:** Renato Mancini Astray

**Affiliations:** 1 Butantan Institute, São Paulo, Brazil

The article is very well presented and clear.

 This work is truly important in the context of combating rabies. My only question about it besides some formatting issues found elsewhere is the absence of a negative serum or a non-neutralizing monoclonal antibody at least to evidence the specificity, which I don't consider was proved here.

 Serum samples of immunized animals or people, particularly those that serum converted in the past and now have little or no potency at all would be valuable to prove that the pseudo particles aren't being blocked by no specific interactions. The use of serum samples is particularly important to make considerations about the use of the methodology in substitution to RFFIT or FAVN as stated in the discussion.

Is the work clearly and accurately presented and does it cite the current literature?

Yes

If applicable, is the statistical analysis and its interpretation appropriate?

Yes

Are all the source data underlying the results available to ensure full reproducibility?

Yes

Is the study design appropriate and is the work technically sound?

Partly

Are the conclusions drawn adequately supported by the results?

Partly

Are sufficient details of methods and analysis provided to allow replication by others?

Yes

Reviewer Expertise:

Viral vaccines

I confirm that I have read this submission and believe that I have an appropriate level of expertise to confirm that it is of an acceptable scientific standard, however I have significant reservations, as outlined above.

# Response

**Published:** 2026-06-22

**Authors:** Eleonora Molesti

**Affiliations:** VisMederi, Not Applicable, Siena, Italy, Italy

We thank the reviewer for this important comment regarding assay specificity. Unfortunately, we did not have access to non-neutralizing monoclonal antibodies or well-characterized negative  sera with prior seroconversion.

 As a negative control, we used a commercially available serum minus preparation from Sigma, which represented the only suitable negative sample available to us at the time of the study. This control consistently showed no inhibition of pseudoparticle entry, supporting the absence of detectable non-specific blocking under our experimental conditions.

 We agree that including additional negative controls, such as non-neutralizing monoclonal antibodies or sera  with negligible neutralizing activity, would further strengthen the demonstration of specificity, and we will consider this in future studies.
